# Effects of thoraco-pelvic supports during prone position in patients with acute lung injury/acute respiratory distress syndrome: a physiological study

**DOI:** 10.1186/cc4933

**Published:** 2006-06-08

**Authors:** Davide Chiumello, Massimo Cressoni, Milena Racagni, Laura Landi, Gianluigi Li Bassi, Federico Polli, Eleonora Carlesso, Luciano Gattinoni

**Affiliations:** 1 Dipartimento di Anestesia e Rianimazione, Fondazione IRCCS – 'Ospedale Maggiore Policlinico, Mangiagalli, Regina Elena', Via F. Sforza 35, 20122 Milan, Italy; 2 Istituto di Anestesia e Rianimazione Università degli Studi di Milano, Via F. Sforza 35, 20122 Milan, Italy

## Abstract

**Introduction:**

This study sought to assess whether the use of thoraco-pelvic supports during prone positioning in patients with acute lung injury/acute respiratory distress syndrome (ALI/ARDS) improves, deteriorates or leaves unmodified gas exchange, hemodynamics and respiratory mechanics.

**Methods:**

We studied 11 patients with ALI/ARDS, sedated and paralyzed, mechanically ventilated in volume control ventilation. Prone positioning with or without thoraco-pelvic supports was applied in a random sequence and maintained for a 1-hour period without changing the ventilation setting. In four healthy subjects the pressures between the body and the contact surface were measured with and without thoraco-pelvic supports. Oxygenation variables (arterial and central venous), physiologic dead space, end-expiratory lung volume (helium dilution technique) and respiratory mechanics (partitioned between lung and chest wall) were measured after 60 minutes in each condition.

**Results:**

With thoraco-pelvic supports, the contact pressures almost doubled in comparison with those measured without supports (19.1 ± 15.2 versus 10.8 ± 7.0 cmH_2_O, *p *≤ 0.05; means ± SD). The oxygenation-related variables were not different in the prone position, with or without thoraco-pelvic supports; neither were the CO_2_-related variables. The lung volumes were similar in the prone position with and without thoraco-pelvic supports. The use of thoraco-pelvic supports, however, did lead to a significant decrease in chest wall compliance from 158.1 ± 77.8 to 102.5 ± 38.0 ml/cmH_2_O and a significantly increased pleural pressure from 4.3 ± 1.9 to 6.1 ± 1.8 cmH_2_O, in comparison with the prone position without supports. Moreover, when thoraco-pelvic supports were added, heart rate increased significantly from 82.1 ± 17.9 to 86.7 ± 16.7 beats/minute and stroke volume index decreased significantly from 37.8 ± 6.8 to 34.9 ± 5.4 ml/m^2^. The increase in pleural pressure change was associated with a significant increase in heart rate (*p *= 0.0003) and decrease in stroke volume index (*p *= 0.0241).

**Conclusion:**

The application of thoraco-pelvic supports decreases chest wall compliance, increases pleural pressure and slightly deteriorates hemodynamics without any advantage in gas exchange. Consequently, we stopped their use in clinical practice.

## Introduction

Prone positioning is used and recommended as a rescue maneuver to improve arterial oxygenation in adult patients with acute lung injury (ALI), acute respiratory distress syndrome (ARDS) [1,2] or chronic obstructive pulmonary disease [3], although its benefits with regard to outcome are not proven [4,5].

Improved oxygenation implies, by definition, improvement of the ventilation/perfusion ratio. This can be achieved through different mechanisms, not mutually exclusive, each documented in the literature: (1) a more uniform distribution of alveolar inflation/ventilation, due to the lower gradient of transpulmonary pressure resulting from the changes in chest wall mechanics, with perfusion being less affected [6-9]; (2) a greater recruitment of the dorsal lung regions in comparison with the derecruitment of the ventral lung regions when changing from the supine to the prone position [10]; (3) an overall increase in end-expiratory lung volume (EELV) as a result of the more favorable position of the diaphragm [11].

Douglas and colleagues [12] used supports under the ribcage and the pelvis of patients with respiratory failure, to prevent their abdomen from bearing the entire weight of the torso. Indeed, some authors have advocated the use of thoraco-pelvic supports to avoid an increase in intra-abdominal pressure, which could limit diaphragm excursion and, consequently, alveolar ventilation in the most dependent lung regions [13,14]. A survey study, in 29 intensive care units, found that thoraco-pelvic supports were routinely applied in 18 of them [15].

However, the use of thoraco-pelvic supports in the prone position has potential drawbacks, such as the possibility of developing pressure sores at the contact surfaces [16]. Because the effectiveness of this intervention is debated, in the present study we set out to investigate whether the use of thoraco-pelvic supports on patients with ALI/ARDS improves, worsens, or has no effect on respiratory mechanics, gas exchange, and hemodynamics.

## Materials and methods

### Study population

Eleven consecutive intubated patients with ALI/ARDS, defined in accordance with standard criteria [17], were included in the study. None of them had a history of chronic obstructive pulmonary disease, heart failure or severe head trauma. Their main clinical characteristics are summarized in Table 1. After completing the study and analyzing the data we realized the possible importance of the contact pressures. We therefore measured the contact pressures directly in four healthy volunteers with or without the thoraco-pelvic supports.

The study was approved by the Institutional Review Board of our hospital. Informed consent, because the patients were incompetent, was obtained in accordance with Italian national regulations (waived consent).

### Study design

**Figure 1:**
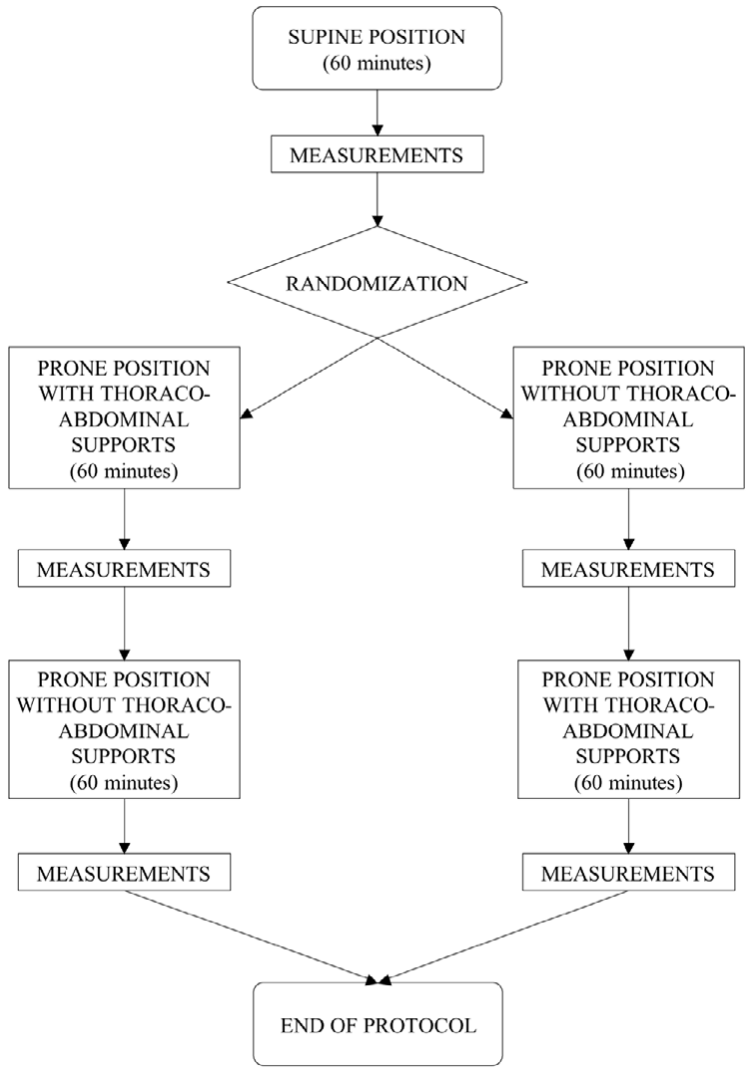
Flow chart of the study protocol.

The patients were first studied in the supine position (1-hour baseline). Subsequently, they were studied in the prone position for 2 hours, for 1 hour with supports and for 1 hour without, in a randomized manner (see flow diagram in Figure 1) for a total duration of 3 hours study time.

**Figure 2:**
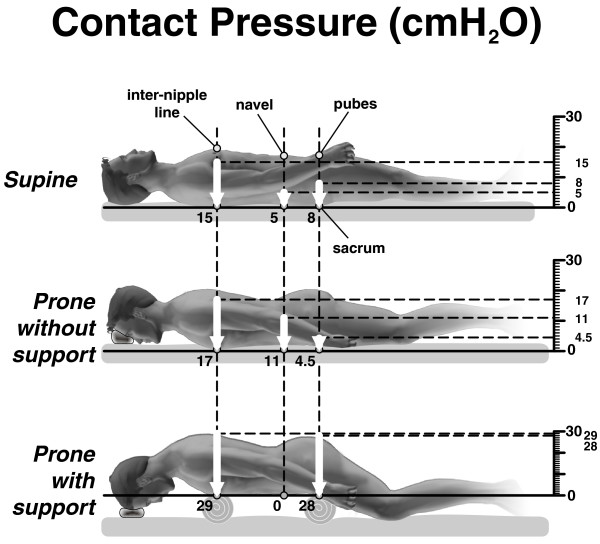
Patients' positions and contact pressures. Patients' positions used in the study: supine (top), prone without supports (center) and prone with thoraco-pelvic supports (bottom). The mean contact pressures (measured with pressure transducers in four healthy volunteers) are also indicated by white arrows. Table 2 shows detailed contact pressures at different sites and global values.

The patients were lying on air-cushioned beds (Total Care^®^; Hill Rom Services Inc., Batesville, IN, USA). In the supine position and in the prone position without supports, the body of each patient was in direct contact with the mattress. In prone position with supports, a roll was placed under the cranial part of the ribcage and a pillow under the pelvic region, so that most of the body weight rested on them. The thoraco-pelvic supports were placed so as to allow free abdominal movements (see Figure 2 and Table 2).

The patients were studied while sedated with fentanyl (1.5 to 5.5 μg/kg per hour) and midazolam (4 to 8 mg/hour), paralyzed with pancuronium bromide (0.05 to 0.1 mg/kg per hour) and ventilated in volume-control mode with a Servo Ventilator 300 C (Siemens, Solna, Sweden). Mechanical ventilation was set by the attending physician on a clinical basis and remained unchanged throughout the study periods. The baseline mean tidal volume was 565.3 ± 160.5 ml (7.2 ± 1.4 ml/kg_IBW_, where IBW stands for ideal body weight; means ± SD), respiratory rate was 17.1 ± 3.5 breaths/minute, inspiratory oxygen fraction was 0.43 ± 0.04, positive end-expiratory pressure (PEEP) was 10.8 ± 1.8 cmH_2_O, and plateau pressure was 22.4 ± 4.3 cmH_2_O.

Fluids, drug infusions and ventilator settings remained unchanged throughout the whole study period.

### Measurements

#### Contact pressures

The pressures between the air-cushioned beds or thoraco-pelvic supports and the body (namely, the contact pressures) were measured in four healthy volunteers (age 28.7 ± 4.9 years, weight 66.2 ± 11.8 kg, body mass index 22.1 ± 2.0 kg/m^2^), in the same three conditions and body positions in which the patients were studied. A plastic bag with a volume of 250 ml containing 100 ml of water and equipped with a pressure transducer (Transpec IV L974; Abbott Ireland, Sligo, Ireland) was used. The zero of the pressure transducer was at the level of the plastic bag. In the supine position, pressure transducers were placed under the shoulders, the lumbar spine, and the sacrum. In the prone position, with and without the thoraco-pelvic supports, pressure transducers were placed in the corresponding positions, under the upper chest, the mesogastrium, and the pelvic region (Figure 2).

#### Gas exchanges and hemodynamics

All variables were recorded at the end of each study period. Blood gas tensions in the arterial and central venous blood were analysed with a blood gas analyzer (IL-1312 Blood Gas Manager; Instrumentation Laboratory, Milan, Italy). Minute metabolic carbon dioxide production, partial pressure of CO_2 _in mixed expired air, and end-tidal concentration of carbon dioxide were measured with a respiratory function monitor (CO_2_SMO™; Novametrix Medical Systems Inc., Wallingford, CT, USA). The venous admixture (estimated from the central venous blood values), the physiological dead space, and the alveolar dead space were computed from standard formulae.

Blood pressures (central and arterial) were measured with disposable pressure transducers (Transpec IV L974) positioned at the mid-axillary line. Cardiac output was measured with the thermo-dilution method, using a Swan–Ganz Oximetry Paceport Thermo-dilution Catheter (Edwards Lifesciences, Irvine, CA, USA) in five patients, and by pulse contour analysis (PiCCO System™ version 4.1; Pulsion Medical System, Munich, Germany) in four. In the five patients with a Swan–Ganz catheter, pulmonary artery and wedge pressures were also recorded. The stroke volume index was computed as the stroke volume divided by the body surface area (BSA). The BSA was obtained with the formula BSA [m^2^] = 0.20247 × height [m]^0.725 ^× weight [kg]^0.425 ^[18].

#### End-expiratory lung volume and respiratory mechanics

EELVs at PEEP were measured with a simplified closed-circuit helium-dilution method, during an end-expiratory pause [19]. An anesthesia bag, filled with 1.5 liters of a known gas mixture (13% helium in oxygen) was connected to the airway opening previously clamped at end-expiration to maintain the PEEP level. Ten manual breaths were subsequently performed. The helium concentration in the bag was then measured with a helium analyzer (PK Morgan Ltd, Chatham, UK) and EELV was computed from the formula EELV = (*V*_i _× [He]_i_/[He]_f_) - *V*_i_, where *V*_i _is the initial gas volume in the anesthesia bag and [He]_i _and [He]_f _are the initial and final concentrations of helium in the bag, respectively.

Airway pressures were measured proximally to the endotracheal tube with a dedicated pressure transducer (MPX 2010 DP; Motorola, Phoenix, AZ, USA). Mean airway pressures were calculated as the area under the airway pressure–time trace, divided by the duration of each breath. Esophageal and gastric pressures were measured with two radio-opaque balloons inflated with 0.5 to 1.0 ml of air (SmartCath; Bicore, Irvine, CA, USA) connected to a pressure transducer (Bentley Trantec; Bentley Laboratories, Irvine, USA). The esophageal and gastric balloons were both positioned in the stomach with the use of an endotracheal tube inserted through the mouth as a guide through the pharynx. The esophageal balloon was then retracted until it reached the upper third of the esophagus. In addition, to ensure the correct position of the catheters, an inspiratory occlusion was made, so that a check for concordant changes in airway, esophageal, and gastric pressures could be made.

Respiratory flow rates were measured with a heated pneumotachograph (Fleisch no. 2; Fleisch, Lausanne, Switzerland) inserted between the proximal tip of the endotracheal tube and the Y-piece of the breathing circuit. Flow and pressure signals were recorded on a personal computer for subsequent analysis with dedicated software (Colligo; Elekton, Milan, Italy). Tidal volumes were obtained by mathematical integration of the measured flow signal. The static compliance of each component of the respiratory system – respiratory system, chest wall, and lung – was calculated as a chord compliance, using standard formulae, with the rapid occlusion method [20]. The end-inspiratory pause button of the ventilator was actioned until airway, esophageal, and gastric pressures decreased from their maximum value to an apparent plateau. Similarly, end-expiratory airway, esophageal, and gastric pressures were recorded after an end-expiratory hold maneuver.

Transpulmonary pressure was computed as the difference between airway pressure and esophageal pressure, and the transdiaphragmatic pressure as the difference between esophageal pressure and gastric pressure. Pleural pressure change, gastric pressure change, and transpulmonary pressure change were calculated as the differences between end-inspiratory and end-expiratory esophageal pressure, gastric pressure, and transpulmonary pressure, respectively.

Intra-abdominal pressure was estimated by measuring the bladder pressure by the method of Cheatham and Safcsak [21].

### Statistical analysis

Data are shown as means ± SD. All data were analyzed with SAS software (version 8.2; SAS Institute, Cary, NC, USA).

The study design included a baseline condition (supine) and two treatments (prone without supports and prone with supports). The treatments were administered to each patient in a randomized order, in accordance with a crossover design.

The effect of the two treatments and of the sequence of their administration was evaluated with an analysis of variance for repeated measures, performed with the SAS MIXED procedure. In addition, each study treatment (prone with and without supports) was compared with baseline (supine) by using paired *t *tests.

To explore the possible association between pleural pressure change and several tested variables, we used the SAS MIXED procedure, building a mixed-effect linear model, in which each patient was treated as a random coefficient. This procedure yielded the parameters of a global regression model, as well as an indication (*p *value) of the significance of the association itself.

## Results

### Contact pressures

Contact pressures recorded in four healthy subjects in the supine and in the prone position with and without supports are summarized in Figure 2. As shown, in shifting the subjects from the supine to the prone position without thoraco-pelvic supports, the contact pressures at thorax and sacrum/pubis did not change significantly, whereas pressures recorded at the abdominal wall surface increased (11.0 ± 1.8 versus 5.8 ± 2.9 cmH_2_O for the supine position). After application of the thoraco-pelvic supports the contact pressures at thorax and pubis increased significantly compared with those in the prone position without supports (29.0 ± 6.5 versus 17.0 ± 7.4 cmH_2_O and 28.3 ± 8.9 versus 4.5 ± 4.2 cmH_2_O, respectively) whereas the contact pressure at the abdominal wall surface was zero because the abdomen remained suspended.

### End-expiratory lung volume and respiratory mechanics

The EELVs and the mechanics of the respiratory system, partitioned into the chest wall and lung components, are summarized in Table 3. Shifting the patients from the supine to the prone position, without supports, led to a decreasing trend of chest wall compliance and to a significant increase in lung compliance. Adding the thoraco-pelvic supports in the prone position led to a further significant decrease in chest wall compliance and a significant increase in pleural pressure. We found no sequence effect (that is, prone after supine or supine after prone; see Figure 1) on lung volumes and respiratory mechanics variables.

### Gas exchange

Table 4 summarizes the gas exchange variables in the supine and in the prone position with and without thoraco-pelvic supports. As shown, the oxygenation-related variables in the arterial and central venous blood improved significantly in shifting the patients from supine to prone without thoraco-pelvic supports. The application of thoraco-pelvic supports did not lead to any further significant change. No significant differences were observed in CO_2_-related variables between the supine and the prone position with or without thoraco-pelvic supports. We found no sequence effect on gas exchange variables.

### Hemodynamics

**Figure 3:**
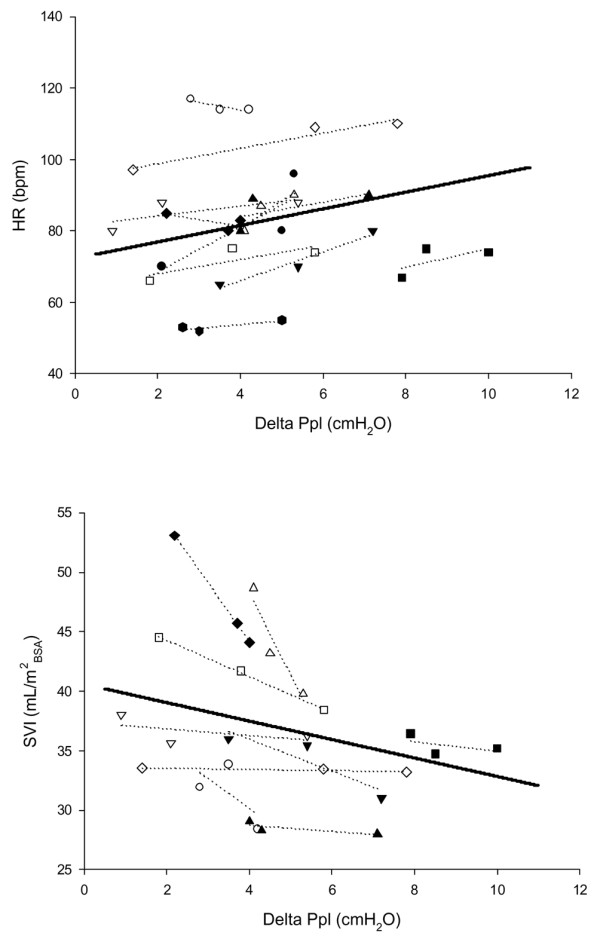
Intrathoracic pressure and hemodynamics. Top panel, association between pleural pressure change (delta Ppl) and heart rate (HR); bottom panel, association between pleural pressure change and stroke volume index (SVI). Each patient is represented with a different symbol and the values recorded in the three different conditions are all indicated, together with the regression line about these points for each individual patient. A regression line for the whole model of association is also depicted (thick line). Pleural pressure change is significantly associated with heart rate (*p *= 0.0003) and with stroke volume index (*p *= 0.0241).

The application of thoraco-pelvic supports caused a significant increase in heart rate and a decrease in stroke volume index and in pulmonary artery pressures, in comparison with the prone position without supports. The other hemodynamic variables (notably cardiac index and systemic vascular resistance) were not affected by the application of thoraco-pelvic supports. There was no sequence effect on hemodynamic variables. We observed a significant association between the level of pleural pressure change and heart rate (*p *= 0.0003) and between pleural pressure change and stroke volume index (*p *= 0.0241) (see Figure 3 and Table 5).

## Discussion

In the present study we found that the prone position with thoraco-pelvic supports, as compared with the prone position without supports, did not affect gas exchange and lung volume but decreased the chest wall compliance, increased the pleural pressure and slightly modified the hemodynamic pattern (heart rate and stroke volume index). In addition, we confirmed the positive effects of the prone position on oxygenation when shifting ALI/ARDS patients from the supine to the prone position, as largely documented in the literature [4,5].

### Mechanics of the respiratory system

When, in previous studies, we directly investigated chest wall displacements by optoelectronic plethysmography we found that both in spontaneously breathing subjects and in paralysed patients with ALI/ARDS in the supine position, the ribcage accounted for about 37% of the chest wall displacement and the abdomen for 63% (that is, the ribcage compliance and abdominal wall compliance were 37% and 63%, respectively, of the whole chest wall compliance) [22,23]. When the subjects were moved to the prone position without pelvic supports, the ribcage accounted for 46.5% of the chest wall displacement, and the abdomen for 53.5% [23]. In experimental animals, too, with a computed tomography scan we found a more even distribution of chest wall displacement [24] when shifting from supine to prone.

If we apply these figures to our actual patients we can estimate that in the supine position the ribcage compliance would have been 86.8 ± 56.3 ml/cmH_2_O and the abdominal wall compliance 148.4 ± 96.2 ml/cmH_2_O (total chest wall compliance 235.2 ± 152.5 ml/cmH_2_O), whereas in the prone position they would have been 73.5 ± 36.2 and 84.6 ± 41.6 ml/cmH_2_O, respectively (total chest wall compliance 158.1 ± 77.8 ml/cmH_2_O). This suggests that, in shifting from supine to prone without thoraco-pelvic supports, the decrease in abdominal wall compliance accounts for most of the decrease in chest wall compliance. If so, the use of thoraco-pelvic supports, which allows free movement of the abdominal wall, should be mostly indicated. In contrast, we found that applying the thoraco-pelvic supports led to a further decrease in chest wall compliance. Thus, at least in patients with ALI/ARDS, when using thoraco-pelvic supports, the possible improvement in abdominal chest wall compliance may be offset by the greater decrease of the ribcage compliance, possibly as a result of the increased contact pressures at the ribcage. In addition, the expected improvement in abdominal wall compliance with thoraco-pelvic supports could be lower than expected because of the greater baseline distension of the unsupported abdominal wall and the possible effects of pelvic supports on the lower abdominal mechanics.

### Lung volumes, gas exchange and hemodynamics

In the present study, as shown previously [4,5], the oxygenation variables increased significantly when the patients were shifted from supine to prone without thoraco-pelvic supports, but did not change when the supports were added. The average EELVs did not change in any position. However, the lung volume increased in some patients after being moved from the supine to the prone position, whereas in others it decreased, suggesting different individual interactions between the opposite effects of the prone position on recruitment (increased lung gas volume) and on increased pleural pressure (decreased transpulmonary pressure). We found that several hemodynamic variables changed significantly between the use and the non-use of supports in the prone position. Although we do not have direct evidence, we speculate that the independent variable that caused the hemodynamic changes is the increase in intrathoracic pressure associated with the use of thoraco-pelvic supports. The hemodynamic changes, in fact, are compatible with the homeostatic response to an initial decrease in effective circulating volume induced by an increase in pleural pressure. The correlation we found between the progressive increase in pleural pressure change and the decrease in stroke volume and increase in heart rate supports this hypothesis.

### Clinical implications

One of the major complications related to the prone position are pressure sores, usually located at the weight-bearing sites such as the bony prominences where the contact pressures are the highest [4,5,25]. A relationship between pressure sores and the duration and magnitude of the contact pressure has been shown [26]. In patients in the prone position, a significantly higher number of new or worsening pressure sores has been found in comparison with the supine position [4,5,27]. The use of thoraco-pelvic supports, by increasing the contact pressures, because of a lower contact surface compared with lying with the body directly on the air-cushioned beds, could potentially increase skin-tissue damage.

## Conclusion

This study suggests that the prone position primarily induces changes in pleural pressure, probably by modifying the geometry and mechanics of the chest wall. Adding the thoraco-pelvic supports does not provide any advantage in oxygenation but increases the pleural pressure. Moreover, although not investigated in this short-term study, increased contact pressures at the interface between the thoraco-pelvic supports and the body may increase, with time, the likelihood of pressure sores. Indeed, in clinical practice, we have stopped using thoraco-pelvic supports in the prone position.

## Key messages

• 	The prone position is a recognized rescue therapy for severe hypoxemia in ARDS.

• 	Allowing free abdominal movement should improve lung mechanics and gas exchange on a theoretical basis.

• 	This hypothesis was tested by studying respiratory mechanics (partitioned into lung and chest wall components), gas exchange and hemodynamics with and without thoraco-pelvic supports.

• 	We could not show any benefit from using thoraco-pelvic supports.

• 	Thoraco-pelvic supports are useless in ARDS patients in the prone position and merely increase the likelihood of pressure sores, as a result of increased contact pressures.

## Abbreviations

ALI = acute lung injury; ARDS = acute respiratory distress syndrome; BSA = body surface area; EELV = end-expiratory lung volume; PEEP = positive end-expiratory pressure.

## Competing interests

LG is a member of the paid KCI Advisory Board. All other authors declare that they have no competing interests.

## Authors' contributions

DC conceived the study, participated in its design and coordination, performed the measurements and wrote a first draft of the manuscript. MC participated in the study design and coordination and performed the measurements. MR participated in the study design and coordination and performed the measurements. LL participated in the study design and coordination and performed the measurements. GLB participated in the study design and coordination and performed the measurements. FP performed the statistical analysis and helped draft the manuscript. EC performed the statistical analysis and helped draft the manuscript. LG conceived the study, participated in its design and coordination, coordinated the final analysis of collected data, and revised the manuscript in writing its final version. All authors read and approved the final manuscript.

## Figures and Tables

**Table 1:** Patients' main characteristics

Patient	Sex (M/F)	Age (years)	Measured weight (kg)	BMI (kg/m^2^)	PEEP (cmH_2_O)	PaO_2_/FiO_2 _(Torr)	Diagnosis	Days of ALI/ARDS	Outcome
1	M	73	75	23,2	9.4	180	Sepsis (from peritonitis)	2	S
2	F	55	55	19,9	10.9	245	Sepsis (from peritonitis)	9	S
3	M	76	85	23,3	8.3	138	Community-acquired pneumonia	4	S
4	M	43	90	23,3	10.6	225	Pneumonia (*ab ingestis*) – sepsis	2	S
5	M	80	70	23,0	11.3	210	Nosocomial pneumonia	13	S
6	M	48	85	24,2	12.8	265	Polytrauma	8	S
7	M	44	80	24,7	9.3	178	Nosocomial pneumonia	6	S
8	M	38	92	23,3	8.8	225	Pneumonia (*ab ingestis*)	7	S
9	M	77	55	22,1	14.0	204	Idiopathic pneumonia in bone marrow transplantation	1	D
10	M	27	80	23,3	12.7	237	Nosocomial pneumonia	4	S
11	M	59	93	23,3	10.3	160	Sepsis	2	S
Overall	10 M, 1 F	56.4 ± 18.0	78.2 ± 13.4	23.1 ± 1.2	10.8 ± 1.81	206.2 ± 38.7	–	5.2 ± 3.7	1D, 10S

BMI, Body mass index; PEEP, positive end-expiratory pressure; PaO_2_/FiO_2_, ratio of arterial oxygen tension to fraction of inspired oxygen; ALI, acute lung injury; ARDS, acute respiratory distress syndrome; S, survived; D, died. Overall results are means ± SD.

**Table 2:** Detailed contact pressures at different sites and global values

Position	Units	Supine	Prone without supports	Prone with supports
Thorax	cmH_2_O	15.4 ± 4.1	17.0 ± 7.4	29.0 ± 6.5^a,b^
Abdomen	cmH_2_O	5.8 ± 2.9	11.0 ± 1.8^a^	0.0 ± 0.0^a,b^
Sacrum/pubis	cmH_2_O	8.0 ± 5.7	4.5 ± 4.2	28.3 ± 8.9^a,b^
Global	cmH_2_O	9.7 ± 5.8	10.8 ± 7.0	19.1 ± 15.2^a,b^

Results are means ± SD. ^a^*p *≤ 0.05 compared with supine; ^b^*p *≤ 0.05 compared with prone without supports.

**Table 3:** Lung volumes and respiratory mechanics

Variable	Units	Supine	Prone without support	Prone with support
Tidal volume (V_T_)	ml	565.3 ± 160.5	577.6 ± 185.3	593.4 ± 200.7
Tidal volume per kg IBW (V_T_/kg_IBW_)	ml/kg	7.2 ± 1.4	7.4 ± 1.6	7.6 ± 1.8
EELV	l	1.12 ± 0.49	1.00 ± 0.26	1.07 ± 0.31
Mean airway pressure	cmH_2_O	15.1 ± 2.1	15.6 ± 2.3	15.7 ± 2.1^a^
Plateau pressure (P_plat_)	cmH_2_O	22.4 ± 4.3	22.1 ± 3.8	23.6 ± 4.5^a,b^
Respiratory system compliance	ml/cmH_2_O	52.1 ± 17.6	52.9 ± 18.8	49.3 ± 18.1^b^
Lung compliance	ml/cmH_2_O	71.5 ± 23.8	93.5 ± 47.3^a^	102.0 ± 47.0^a^
Chest wall compliance	ml/cmH_2_O	235.2 ± 152.5	158.1 ± 77.8	102.5 ± 38.0^a,b^
Transpulmonary pressure change^c^	cmH_2_O	8.4 ± 2.2	7.1 ± 2.2^a^	6.6 ± 2.3^a^
Pleural pressure change^c^	cmH_2_O	3.2 ± 1.9	4.3 ± 1.9	6.1 ± 1.8^a,b^
Gastric pressure	cmH_2_O	13.4 ± 4.0	14.3 ± 3.5	13.2 ± 4.3
Gastric pressure change^c^	cmH_2_O	2.6 ± 0.8	3.4 ± 1.1^a^	4.5 ± 1.9^a,b^
Transdiaphragmatic pressure	cmH_2_O	0.6 ± 2.0	0.9 ± 1.6	1.6 ± 1.7
Bladder pressure	cmH_2_O	12.0 ± 2.8	14.5 ± 3.4^a^	14.5 ± 3.7^a^

Results are means ± SD. IBW, ideal body weight; EELV, end-expiratory lung volume. ^a^*p *≤ 0.05 compared with supine; ^b^*p *≤ 0.05 compared with prone without supports; ^c^difference between end-inspiration and end-expiration

**Table 4:** Gas exchanges

Variable	Units	Supine	Prone without supports	Prone with supports
PaO_2_/FiO_2_	Torr [kPa]	206.2 ± 38.7 [27.5 ± 5.2]	261.8 ± 41.2^a ^[34.9 ± 5.5]	265.0 ± 40.0^a ^[35.3 ± 5.3]
PaO_2_	Torr [kPa]	87.7 ± 10.2 [11.7 ± 1.4]	112.5 ± 16.0^a ^[15.0 ± 2.1]	113.4 ± 12.0^a ^[15.1 ± 1.6]
SaO_2_	%	95.7 ± 1.0	96.6 ± 0.6^a^	96.7 ± 0.6^a^
PvO_2_	Torr [kPa]	44.6 ± 4.0 [5.9 ± 0.5]	49.0 ± 6.3^a ^[6.5 ± 0.8]	47.7 ± 5.6^a ^[6.4 ± 0.7]
SvO_2_	%	77.2 ± 4.4	80.3 ± 5.4^a^	79.6 ± 5.7^a^
pH_a_		7.37 ± 0.06	7.37 ± 0.05	7.36 ± 0.06
Ve	l/minute	9.4 ± 2.5	9.6 ± 2.8	9.9 ± 3.1
PaCO_2_	Torr [kPa]	43.9 ± 4.2 [5.9 ± 0.6]	43.6 ± 4.2 [5.8 ± 0.6]	44.3 ± 6.1 [5.9 ± 0.8]
PvCO_2_	Torr [kPa]	51.0 ± 6.6 [6.8 ± 0.9]	52.1 ± 6.8 [6.9 ± 0.9]	52.2 ± 6.4 [7.0 ± 0.9]
pH_v_		7.35 ± 0.05	7.34 ± 0.06	7.34 ± 0.05
VCO_2_	ml/minute	145.5 ± 38.8	143.3 ± 40.9	140.4 ± 42.9
V_d_/V_t_		0.62 ± 0.10	0.63 ± 0.11	0.63 ± 0.13
V_d_/V_t_(alv)		0.18 ± 0.15	0.19 ± 0.15	0.18 ± 0.16

Results are means ± SD. PaO_2_/FiO_2_, ratio of arterial oxygen tension to fraction of inspired oxygen; SaO_2_, arterial oxygen saturation; PvO_2_, mixed-venous oxygen tension; SvO_2_, mixed-venous oxygen saturation; pH_a_, arterial blood pH; Ve, minute ventilation; PaCO_2_, arterial carbon dioxide tension; PvCO_2_, mixed-venous carbon dioxide tension; pH_v_, venous blood pH; VCO_2_, minute metabolic carbon dioxide production; V_d_/V_t_, dead space; V_d_/V_t_(alv), alveolar dead space. ^a^*p *≤ 0.05 compared with supine.

**Table 5:** Hemodynamics

Variable	Units	Supine	Prone without supports	Prone with supports
CI	(l/minute)/m^2^_BSA_	3.2 ± 0.7	3.2 ± 0.6	3.1 ± 0.5
SVI	ml/m^2^_BSA_	38.1 ± 7.2	37.8 ± 6.8	34.9 ± 5.4^a,b^
HR	min^-1^	79.6 ± 17.3	82.1 ± 17.9	86.7 ± 16.7^a,b^
Mean BP	mmHg	83.7 ± 8.0	89.2 ± 9.4	85.2 ± 11.8
Systolic BP	mmHg	123.0 ± 16.5	132.0 ± 18.4^a^	123.8 ± 19.8
Diastolic BP	mmHg	64.0 ± 7.6	67.8 ± 9.8	65.9 ± 9.8
Mean PAP^c^	mmHg	27.4 ± 3.0	31.0 ± 6.6	27.1 ± 9.0^b^
Systolic PAP^c^	mmHg	38.6 ± 5.1	41.8 ± 11.3	36.8 ± 11.9^b^
Diastolic PAP^c^	mmHg	21.8 ± 1.9	25.6 ± 4.7	22.2 ± 7.5^b^
WP^d^	mmHg	22.0 ± 5.7	24.4 ± 6.6	23.2 ± 7.5
CVP	mmHg	12.0 ± 2.3	13.0 ± 2.8	12.7 ± 4.3
Diuresis	ml	111 ± 71	90 ± 64^a^	91 ± 58^a^

CI, cardiac index; BSA, body surface area; SVI, stroke volume index; HR, heart rate; BP, arterial blood pressure; PAP, pulmonary artery pressure; WP, wedge pressure; CVP, central venous pressure. ^a^*p *≤ 0.05 compared with supine; ^b^*p *≤ 0.05 compared with prone without supports; ^c^cardiac output in nine patients only; ^d^Swan–Ganz in five patients only.
